# Food Matrix Effects on Plant-Derived Bioactive Compounds and Micronutrients: Implications for Functional Food Development

**DOI:** 10.3390/ijms27125503

**Published:** 2026-06-18

**Authors:** Patroklos Vareltzis, Smaro Kyroglou, Evangelia Pasidi, Georgios Oikonomou, Thetis Gkogkou, Maria Govari, Konstantinos Kalogiannis, Olga Gortzi

**Affiliations:** 1 Chemical Engineering Department, Aristotle University of Thessaloniki, 54124 Thessaloniki, Greece; kyrosmar@cheng.auth.gr (S.K.); egpasidi@cheng.auth.gr (E.P.); goikonoa@cheng.auth.gr (G.O.); thetida.gk@gmail.com (T.G.); 2 POSS-Driving Innovation in Functional Foods IKE, 54640 Thessaloniki, Greece; ogortzi@gmail.com; 3 Department of Food Science and Technology, University of Peloponesse, 24100 Kalamata, Greece; m.govari@uop.gr; 4 Chemical Engineering Department, University of Western Macedonia, 50150 Kozani, Greece; kkalogiannis@uowm.gr; 5 Department of Agriculture Crop Production and Rural Environment, School of Agriculture Sciences, University of Thessaly, 38446 Volos, Greece

**Keywords:** food matrix effects, bioaccessibility, plant bioactives, micronutrient bioavailability, functional food design

## Abstract

Even though the functional food market has rapidly increased in recent years, the links between bioactive-rich formulations and consumers’ health benefit are not fully established, mainly because of insufficient consideration of food matrix effects. This review provides a comprehensive and integrated evaluation of how food matrix properties (structural and physicochemical) affect the bioaccessibility of plant bioactive compounds. Unlike many reviews that focus on a single nutrient approach, we highlight quantitative evidence of how bioaccessibility can be affected by matrix properties, illustrating the interactions between main food components (lipids, proteins, dietary fiber and minerals). This review integrates fragmented information among different areas of food and nutrition sciences, i.e., food structure, gastrointestinal science, mineral chemistry, protein chemistry, providing a holistic framework for Quality by Design (QbD) functional food development. Synergisms and antagonistic behaviors, threshold effects, and concentration-dependent behaviors are analyzed comparatively for the most common plant-derived bioactives, such as polyphenols, carotenoids, curcuminoids and minerals (iron, zinc and calcium). We propose a matrix-informed optimization as a prerequisite for credible health claims and sustainable plant-based nutrition strategies. This can ultimately serve as a foundation for next-generation functional food development based on bioaccessibility, supporting the central argument *that functional food development should move from composition-based fortification to bioaccessibility-based matrix engineering*.

## 1. Introduction and Fundamentals of Food Matrix Effects

The growing awareness among consumers that diet plays an important role in the prevention and management of chronic diseases, as well as generally in their wellness, is reflected in the rapid rise of the functional food sector. The health-promoting effects of foods fortified with plant-derived bioactive ingredients and minerals, such as polyphenols, carotenoids, vitamins, zinc and others, are increasingly marketed worldwide [1,2]. However, there is a persistent missing link between the presence of the added bioactive compounds in the foods and their actual physiological impact [3]. The mere presence of a bioactive ingredient alone is not enough to predict physiological effects. Growing evidence suggests that the matrix and its physicochemical properties and structure determine to a great extent the release and absorption of bioactive compounds during gastrointestinal digestion [4].

The approach of treating added nutrients as isolated entities in foods has been challenged by the increased evidence of the effects that the food matrix itself can exert on the final bioactivity [5,6]. The food matrix is a complex, hierarchical structure of networks composed of basic food components, such as proteins, lipids, carbohydrates, fibers, minerals and water. Many dynamic processes take place during gastrointestinal digestion, such as matrix disintegration, enzymatic and non-enzymatic hydrolysis, emulsification and phase transitions. Bioaccessibility, i.e., the fraction of a compound released from matrix and transformed into absorbable forms, as well as bioavailability are mainly governed by the aforementioned processes. Therefore, identical compounds may have a totally different metabolic fate depending on matrix composition, processing history and co-ingested ingredients [4,6,7].

The interactions between matrix and bioactive compounds have been extensively researched. However, the field remains fragmented, since most of the original research studies and reviews focus on specific families of compounds (e.g., polyphenols or carotenoids) or isolated optimization strategies, such as encapsulation [8,9]. Information on a unified approach, taking into account the interplay between matrix structure and composition, digestive processes, compound interactions and functional outcomes, is scarce. This limitation results in the lack of rational functional food design with systematic optimization of matrix structure, leading to reduced efficacy and weakened health claims.

The need for a unified, more holistic approach in functional food development is further strengthened by recent trends in consumer behavior. The growing adoption of plant-based diets raises concerns about the adequate absorption of essential nutrients, since plant matrices often contain absorption inhibitors, i.e., phytates, oxalates and others [10,11,12]. Understanding the inherent matrix antagonisms and potential enhancers is of crucial importance to achieve adequate iron, zinc and calcium uptake in plant-derived foods. Furthermore, the data on the advancement in digestion engineering, cell cultures and intestinal transport remain scattered across various scientific fields. Finally, a variety of tools have been developed to improve delivery of functional ingredients, such as fermentation, nanoencapsulation, structured lipids and others [13,14,15,16,17,18]. However, most of these innovations have been tested on laboratory food systems, while their success depends on the compatibility with real food matrices undergoing real digestion processes.

Additionally to the above-mentioned limitations and gaps, recent publications and similar reviews [3,7,19] address these aspects separately or focus on single dimensions (processing, methodology or compound classes). The contribution of the present review is to integrate these fragmented perspectives into a matrix-informed framework for functional food development. Emphasis is placed on quantitative examples, threshold effects, and compound-specific mechanisms that are directly relevant to formulation design. Specifically, its objectives are: (i) to integrate current knowledge on how proteins, lipids, dietary fibers, and minerals influence the release and solubilization of polyphenols, carotenoids, curcuminoids, and key minerals; (ii) to identify quantitative thresholds and complex interactions—such as critical phytic acid–mineral molar ratios and concentration-dependent polyphenol effects—that determine shifts in bioaccessibility; and (iii) to propose rational design principles for functional food development based on mechanistic evidence. This review addresses the missing link between food fortification and functional efficacy by combining mechanistic and practical approaches to optimize matrix architecture for credible health claims and sustainable nutrition strategies based on plant-derived food systems.

## 2. Food Matrix Composition and Its Impact on Bioactive Release

### 2.1. Structural Organization and Bioactive Sequestration

The bioavailability of food bioactive compounds is inherently determined by the composition and structural organization of the food matrix. Matrix effects are dictated largely by the chemical components within foodstuffs, including lipids, proteins, carbohydrates, minerals and others [7]. The physicochemical properties of these components influence the composition of the gastric and intestinal fluids during digestion and can modulate the solubility, metabolism and uptake of the co-ingested dietary nutrients like phytochemicals [20]. There are several examples of this in the literature. In the case of quercetin, the food matrix and glycosidic form strongly influence absorption. Pure quercetin-3-rutinoside and quercetin from apples exhibited lower bioavailability compared to onion-derived quercetin, which is rich in quercetin glucosides, indicating that both matrix composition and chemical form determine intestinal uptake [21,22]. Similar antagonistic behavior has been observed in the case of zinc and phytate-rich matrices. Food preparations increasing the phytic acid content decrease zinc bioavailability, while the extent of the decrease depends also on the source of the zinc (inorganic, bisglycinate, proteinate), as well as on the molar ratio of Zn to phytic acid; a significant impact is noted at molar ratios higher than 1:100 (phytic acid to zinc) [23,24,25]. Contrary to this antagonistic behavior, curcumin from turmeric demonstrates the opposite matrix effect: co-digestion with dietary lipids leads to a 4-to-5-fold enhancement of bioavailability compared to when consumed alone in aqueous solution. Potentially, the lipid matrix facilitates micellarization of this lipophilic compound [26,27]. It can be deduced from the above that simple nutrient quantification is not enough to safely predict the functional efficacy of the food.

Another critical role of the food matrix is its hardness. The higher the hardness, the more time is needed for mastication with increased force. These conditions (prolonged mastication time and higher mechanical force) enhance the release of the bioactive compounds present in the food matrix [7]. The solubilization of phytochemicals into saliva during oral processing is also influenced by the food matrix composition, with liquid-rich matrices promoting greater dilution and initial bioactive release.

Matrix structure has a profound effect also on carotenoid bioaccessibility in carrots: β-carotene absorption from carrot juice was 2.33 times higher than from consuming raw carrots [28], while the in vitro release of carotenes was highest at ~34% for the cooked carrot puree with the smallest cell wall particles (d(0.5) = 70 μm) compared to ~29% blanched carrot puree, which had the largest plant cell cluster particles with d(0.5) = 200 μm, illustrating how fiber matrix structure can influence the release of the bioactive compound [29].

The structural thresholds where a matrix shifts from a barrier to a facilitator of bioactive release are not defined in most available studies. Therefore, future research should focus on quantifying the bioaccessible fraction of plant bioactives in relation to matrix architecture and oral and gastrointestinal disintegration. In order to have comparable results across different research groups, these studies should use standardized digestion protocols and real food systems rather than simplified model matrices.

The effects of major food matrix components on the release and bioaccessibility of plant-derived bioactives and micronutrients are summarized in Table S1 (Supplementary Material).

### 2.2. Protein-Bioactive Interactions

Proteins are one of the most abundant components in food matrices. They can exhibit either enhancing or inhibitory effects on the bioaccessibility of bioactive substances, depending on the chemical class the bioactive belongs to. The bioactives can bind to the proteins via both covalent and non-covalent interactions, forming new complexes with variable solubility. These new complexes may alter digestibility and/or bioavailability, since they do not retain the physicochemical properties of the original compounds [30,31]. In the case of functional foods and nutraceuticals, this interaction is critical because many products are fortified with proteins and phytochemicals. However, studies suggest that these proteins may reduce the absorption of phytochemicals, diminishing their health benefits. The interaction between proteins and bioactives must therefore be carefully considered in functional food formulation.

Several protein-bioactive interactions have been studied, showing their bidirectional nature. Carotenoid bioavailability is enhanced by β-lactoglobulin (β-Lg), which binds hydrophobic compounds and transports them to the brush border membrane of enterocytes [32]. Recent research showed that addition of β-Lg to test systems increased carotenoid bioaccessibility after thermal processing by 26% to 354%, depending on the temperature (25 to 121 °C), compared to controls without protein [33]. On the contrary, green tea catechins’ bioaccessibility was hindered by β-casein (β-CN). Recent research showed that epigallocatechin gallate (EGCG) and epigallocatechin (EGC) bioaccessibility was reduced in the presence of β-CN, while epicatechin (EC) bioaccessibility was increased [34]. Furthermore, in emulsions containing EGCG stabilized by sodium caseinate, a 64% enhancement in EGCG bioaccessibility was observed compared to free EGCG after simulated gastrointestinal digestion. It was noticed that sodium caseinate facilitated higher intestinal permeability of EGCG across Caco-2 monolayers. This was attributed to strong intermolecular interactions between caseins and EGCG that promote bioavailability [35]. These results support the view that the proteins’ effects are species specific and not universal [36].

Similarly, proteins exhibit species-specific effects on mineral bioaccessibility. Researchers have shown that soy protein significantly enhanced zinc bioaccessibility from sorghum and rice in raw and cooked grain by 50 and 90%, respectively. However, identical protein addition decreased iron bioaccessibility in both grains, verifying the variability of protein effects on minerals’ bioaccessibility [37]. However, this decrease can be attributed to the phytic acid and a protein moiety present in soy protein [38]. This finding highlights the complexity of food component interactions and the need to decouple the effects of different types of compounds to understand their true effects on bioactives’ bioavailability and bioaccessibility.

Protein facilitation was further demonstrated in whey protein–mineral complexes. When whey proteins were digested and then incubated with minerals, the digested whey proteins enhanced mineral bioavailability and cellular uptake in Caco-2 intestinal cell models compared to free minerals alone. It was suggested that protein hydrolysis products can protect and facilitate mineral absorption [39,40]. Protein hydrolysates can increase the solubility of certain minerals, i.e., via chelation, and act as vehicles for transportation across the intestinal lumen [41].

Protein structure and processing-induced modifications to bioactive release, complex stability, intestinal transport, and cellular uptake are not yet well defined. Researchers have at their disposal new tools, i.e., peptidomics, digestion kinetics and binding thermodynamics, that could be integrated to distinguish (and predict) whether proteins can act as protective carriers or as inhibitors of bioactives.

### 2.3. Dietary Fiber and Polysaccharide Interactions

Polyphenols and other bioactive compounds are often bound to the various dietary fibers present in the food matrix [42,43]. To be released during gastrointestinal digestion, extra steps are required; this results in decreased release and bioaccessibility compared to unbound polyphenols. The insoluble fraction of dietary fibers might also contain highly polymerized phenolic species such as tannins and lignins that have low bioaccessibility due to limited release from the matrix [44,45].

However, the relationship between dietary fiber and bioactive bioaccessibility is not uniform. While dietary fiber (such as hemicellulose) generally causes detrimental effects on polyphenol bioaccessibility, the presence of viscous and protein-rich meals is likely to cause additional reductions. Dietary fibers may interact with polyphenols and carotenoids at the digestive tract, and in some cases, this interaction might improve compound bioaccessibility in the digestive tract, and hence their bioactivities [46,47]. Insoluble fibers like cellulose and hemicellulose primarily reduce bioaccessibility through non-covalent sequestration, whereas soluble fibers like β-glucans and pectins can enhance bioavailability by modifying intestinal pH and microbiota populations, demonstrating that fiber type determines whether inhibition or facilitation occurs. This contradictory behavior highlights the importance of understanding how specific fiber types interact with specific bioactive components.

Quercetin and other polyphenols can interact with apple fiber and plant cell wall polysaccharides, which may alter their release, solubility, and bioaccessibility during digestion. Research has shown that quercetin has higher affinity toward apple fiber than citrus fiber. This can be attributed to the fact that apple fiber contains pectins that promote hydrogen bonding and hydrophobic interactions with quercetin, such as homogalacturonan regions and neutral sugar side chains [48].

Indeed, FTIR-ATR analysis indicated structural changes after quercetin binding [49]. Fiber constituents (cellulose, hemicellulose, pectin) can give rise to non-covalent complexes resistant to enzymatic hydrolysis [49,50]. Similarly, arabinoxylans and other hemicelluloses greatly inhibited bioaccessibility of ferulic acid (<1%) in intact grain products; on the contrary, when free ferulic acid was added to flour, bioaccessibility increased to 60% [51].

The amount of pectin in food matrices was not proportional to carotenoid micellarization. Micellarization of free carotenoids was favored by the high degree of methyl esterification (DM) in pectin, while micellarization of less polar carotenoids was influenced by the molecular weight of the pectin [52,53,54]. For citrus pectin-based emulsions containing β-carotene-enriched oil, the β-carotene bioaccessibility was enhanced for higher DM pectin compared to emulsions with pectin with a lower DM, showing that the DM plays an important role [55].

These findings indicate that dietary fiber does not act as a universal inhibitor of bioaccessibility, rather than a matrix-dependent modulator. The chemical structure of the fiber and the physicochemical properties of the bioactive are the two main factors governing the effect of dietary fiber, as was illustrated with the contrasting behaviors of quercetin, ferulic acid and carotenoids in different fiber-rich systems.

### 2.4. Lipid Matrix Effects and Lipophilic Compound Solubilization

When dietary lipids are not sufficient, even bioactives with high intrinsic lipophilicity may fail to form the nanoscale micellar particles required for transcytosis across the intestinal epithelium [56]. The optimal lipid content varies by bioactive class: The amount of dietary fat required to ensure carotenoid absorption is relatively low (~3–5 g per meal), although it depends on the physicochemical characteristics of the carotenoids ingested [57].

On the contrary, curcumin may benefit from higher lipid levels. Curcumin absorption increased nearly 2.5-fold when consumed with 20% lipid content such as coconut oil, demonstrating that adequate lipid incorporation dramatically enhances polyphenol bioavailability [58]. The mechanism underlying this enhancement involves lipase-mediated hydrolysis of dietary triglycerides (TAGs) into monoacylglycerols (MAGs) and free fatty acids (FFAs), which integrate with phospholipids from bile salts to form mixed micelles (typical diameter 2–10 nm) capable of solubilizing hydrophobic bioactives [59]. Lipid concentration had an even more pronounced effect on β-carotene bioaccessibility, which showed a remarkable 93.2% bioaccessibility when corn oil was added at 10% concentration [60].

In addition to the lipid content, the type of lipids also plays a significant role in bioaccessibility by facilitating the formation of micelles of specific bioactive classes. Medium-chain triglycerides (MCTs) form less stable mixed micelles than long-chain triglycerides (LCTs) [61]. Together with the type of lipids, the degree of unsaturation can significantly influence the rate of lipolysis and, therefore, the subsequent micelle formation kinetics [62,63]. Longer and more saturated chains resulted in a slower initial rate and lower degree of lipolysis in the small intestine. However, greater bioaccessibility of curcumin was observed only with longer chains [64]. On the other hand, curcumin in linseed oil (n-3 rich oil) showed higher levels of curcumin in serum, liver, heart and brain compared to curcumin incorporated into coconut oil (medium-chain fatty acid rich) or sunflower oil (n-6-rich nanoemulsions) [65].

Fat-soluble vitamin D3 is another example of how both the concentration and the type of lipid can affect bioavailability. It was shown that bioavailability of vitamin D3 increased 32% when consumed with a meal containing 30% fat compared to minimal lipid content, while the content in mono- or poly-unsaturated fatty acids did not affect the vitamin’s absorption [66]. Furthermore, LCTs were more effective than MCTs during intestinal absorption at increasing vitamin D bioaccessibility. Long-chain fatty acids form mixed micelles (micelles and vesicles) that have larger non-polar regimes capable of accommodating large lipophilic bioactive molecules. Conversely, large lipophilic bioactives cannot easily be accommodated into the smaller non-polar regimes found in mixed micelles formed by medium-chain fatty acids [67]. Similar behavior was observed in the case of CoQ10, with its bioavailability increasing 1.8–2.8-fold when the compound was incorporated into lipid-based formulations (nanoemulsions or beverage systems) compared to CoQ10 dissolved in oil alone [68].

## 3. Bioaccessibility of Specific Plant Bioactives and Micronutrients

Section 2 analyzed how major matrix components affect the release, solubilization, protection or sequestration of bioactive components during digestion. This section (Section 3) applies this matrix-based framework to specific classes of plant-derived bioactives and micronutrients. In this way, the general mechanisms described previously can be interpreted in terms of the physicochemical properties of each compound class, including polarity, molecular size, glycosylation, degree of polymerization, mineral speciation, and dependence on micellarization or microbial transformation. The following subsections move from matrix mechanisms to compound-specific bioaccessibility outcomes, highlighting why the same food matrix may enhance one bioactive while inhibiting another.

### 3.1. Polyphenols and Flavonoids

Polyphenols represent the largest class of plant bioactives, and their bioaccessibility varies considerably based on both their chemical structure and the surrounding food matrix. The bioavailability of polyphenols is ranked according to chemical class as follows: phenolic acids > isoflavones > flavonols > catechins > flavanones > proanthocyanidins > anthocyanins [69]. This ranking reflects both structural properties that influence release from food matrices and the inherent metabolic stability of each class during gastrointestinal transit. After reviewing 97 bioavailability studies, Manach et al. (2005) [70] concluded that the most well-absorbed polyphenols were gallic acid and isoflavones, followed by catechins, flavanones, and quercetin glucosides, but with different kinetics. The least well-absorbed polyphenols were the proanthocyanidins, the galloylated tea catechins, and the anthocyanins [70].

The relationship between polyphenol structure and food matrix interactions is particularly significant for anthocyanins. Quantitative evidence illuminates the magnitude of these structural differences: phenolic acids demonstrate approximately 60–75% release during the intestinal digestion phase in complex food matrices, while anthocyanins, the most structurally complex—show only 8–20% under identical conditions [71,72,73]. This 3–6-fold difference reflects the structural complexity of the polyphenol backbone: phenolic acids (simple carboxyl-substituted benzene rings) require minimal enzymatic modification for absorption, whereas anthocyanins (glucosylated flavonoid structures with unstable glycosidic bonds) undergo extensive degradation and metabolism during gastrointestinal transit, reducing their bioaccessible fraction [74,75]. The significantly reduced bioaccessibility of proanthocyanidins can be attributed to their degree of polymerization; Zeng et al. (2024) showed that the bioaccessibility of the polymerized forms was only 5–10% of that of monomeric flavan-3-ols [76].

From a mechanistic point of view, the bioavailability ranking reflects three primary pathways: (1) Molecular weight effect: Larger polyphenols (proanthocyanidins > 3000 Da) cannot pass through paracellular pathways and must be transported transcellularly via limited transporters; (2) hydroxylation pattern: Catechols (e.g., catechins with three-OH configuration) are more susceptible to oxidation and polymerization than gallates or other patterns; (3) glycosylation: Microbial β-glucosidase activity may contribute to anthocyanin variability, together with differences in matrix release, pH stability, intestinal transport, and microbiota composition, causing significant (up to ~10 fold) interindividual variation in anthocyanin bioavailability [77,78].

### 3.2. Carotenoids and Lipophilic Bioactives

The structural organization of carotenoids within chromoplasts plays a critical role in their release from plant cell structures. The mechanism underlying this cell wall effect involves multiple barriers: (1) Physical encapsulation of carotenoids within crystalline carotenoid–protein complexes in chromoplasts; (2) β-1,4 and β-1,6 linkages in hemicellulose forming hydrogen-bonded networks that prevent solvent access; and (3) lipid barriers (plastid membranes) surrounding carotenoid-rich chromoplast structures [79,80].

Quantitative evidence reveals β-carotene bioaccessibility is strongly matrix dependent and generally increases with mechanical disruption, heat treatment, and the presence of dietary lipids. Heat treatment can significantly modify carotenoid bioavailability—the in vitro bioaccessibility of all carotenoids was significantly higher (37.6 to 39.5%) in pasteurized juices than in freshly squeezed juices [81]. This improvement reflects heat-induced disruption of cellular structures that release carotenoids and facilitate their solubilization in food lipids. González et al. (2018) [82] used tomato products mixed with 5% olive oil and found β-carotene bioaccessibility of 14.1% in untreated tomato-derived product, increasing up to 21.6% under the best pulsed-electric-field condition [82]. Tomaş et al. (2019) [83] found that carotene’s bioaccessibility improved ~5-fold by the effect of cooking and addition of extra virgin olive oil to the samples compared to control tomato sauce without oil and cooking [83].

### 3.3. Minerals

Minerals differ from the previously described bioactive classes in that they are inorganic ions often found as salts, or in complexes with amino acids, organic acids, or peptides. Generally, they have relatively low bioavailability and are quite often bound by antagonists such as phytate, polyphenols, fiber and other constituents in plants [84]. Two main mechanisms affecting their bioavailability are chelation and coprecipitation [85]. The quantitative relationships between mineral antagonists and target minerals are critical for functional food development.

#### 3.3.1. Iron

Many studies have demonstrated that calcium could inhibit iron absorption on a short-term basis, while long-term calcium supplementation has no effect on the overall iron homeostasis. This has been attributed to the transient and adaptive nature of iron absorption [86]. Calcium has been shown to inhibit iron absorption in short-term single-meal studies. Iron absorption was reduced by approximately 50–60% when administered with 300–600 mg of calcium, while similar inhibition was observed when 165 mg of calcium was provided as milk, cheese, or calcium chloride [87]. The simultaneous presence of calcium and iron in the intestinal lumen seems to cause this inhibitory effect, which has been attributed mainly to mucosal transfer or intestinal transport rather than to a simple fixed Ca:Fe molar ratio effect [88]. In another study, administration of calcium and iron at molar ratios between 500 and 1000:1 increased the uptake of non-heme iron and decreased its efflux [89]. The molecular mechanisms involved may include modulation of iron transporters by calcium, but more research is needed to elucidate this process. Furthermore, there are few studies on how iron affects calcium absorption.

The most impactful iron antagonist is phytic acid (myo-inositol hexakisphosphate, IP6). At intestinal pH, its six phosphate groups form highly stable and insoluble ferric-phytate complexes that limit the iron’s bioavailability [90,91]. Iron bioavailability dropped by approximately 70%, because of phytic acid concentrations found in wholegrain cereals [92]. Despite similar total iron content, iron bioavailability can be substantially high from refined or low-phytate cereal products compared to whole-grain, phytate-rich ones.

Wheat bran reduced geometric mean iron absorption from white flour cereal from 11.6% to 4.3% [93]. Inversely, complete phytate degradation in cereal/legume porridges increased iron absorption up to 12-fold, from 0.99% to 11.54% in human isotope studies [94]. Under low-inhibitor reference conditions, modeled or adjusted non-heme iron absorption can approach ~40%, particularly in iron-depleted individuals or meals containing enhancers such as ascorbic acid [94]. Another major antagonism is the interactions between polyphenols and iron. Delimont et al. (2017) [95] reviewed several such interactions and showed that catechin-rich beverages reduced iron bioavailability significantly at neutral pH. On the contrary, beverages with lower polyphenol content exhibited a 35 to 40% iron bioavailability [95]. The extent of iron bioaccessibility inhibition varies dramatically by polyphenol type: In in vitro experiments, low dose quercetin supplementation increased apical uptake but decreased efflux [96]. Purified EGCG at 150–300 mg reduced non-heme iron absorption by ~14–27% in humans [97], while whole green tea matrices with high total polyphenol content can inhibit iron absorption much more strongly [98]. Fe(III)–EGCG complexes can be formed with different constants (log K = 4.39, 4.78, and 3.93) for different stoichiometries at pH 7.4 [99]. However, the role of polyphenols in iron metabolism remains poorly understood, with contradictory results being reported in the literature. In some model systems, polyphenols may enhance iron stability through protein–polyphenol–iron interactions, but this effect appears to be dose, structure, and matrix dependent [100,101]. Others report reduced iron bioavailability under gastrointestinal conditions due to the formation of insoluble iron complexes [102,103]. These seemingly contradictory findings may reflect differences in polyphenol types and amounts used in different studies, with some types enhancing absorption, others having neutral effects, and others acting as inhibitors [104,105]. The presence of proteins in the food matrix makes things even more complicated. Understanding how polyphenols interact with protein–iron complexes is therefore essential for improving iron fortification strategies. However, little is known about how dietary polyphenols affect the behavior of these complexes [101].

Mineral iron absorption could potentially be enhanced with ascorbic acid (AA—vitamin C) through two distinct mechanisms: (1) Formation of soluble 1:1 or 1:2 ascorbic acid–iron chelation complexes that resist precipitation at the elevated pH (6–7) at the intestinal phase where absorption occurs [106,107,108], and (2) reduction of ferric iron (Fe^3+^) to the more absorbable ferrous form (Fe^2+^) via donation of electrons across the gastric pH gradient [109,110,111]. Ascorbic acid doses up to 75 mg/100 g food in a large wheat–chapati fortification study reported significantly enhanced iron bioaccessibility, with strong concentration-dependent effects [112]. Wheat flour fortified with AA showed approximately double predicted iron bioavailability compared with controls lacking AA [113]. These results are also backed up by human meal studies. Raising AA from low molar ratios to ~70 mg per meal significantly increased iron absorption, from 5.1% to 8.2%, an approximate 60% relative increase [114]. Earlier Derman-type infant cereal studies reported 2–3-fold increases at AA:Fe molar ratios comparable to 25–75 mg of AA per meal [115].

Table 1 contains indicative recent human trial studies illustrating the above-mentioned interactions and specific components effects on iron absorption.

Results in Table 1 confirm that phytic acid has the clearest quantitative inhibitory effect in human trials, while polyphenols significantly reduce the solubility of iron, having a detrimental effect on iron absorption. Calcium on the other hand, exerts a small but significant acute inhibitory effect.

#### 3.3.2. Zinc and Magnesium

Another potential mineral interaction involves magnesium, zinc, and phytate in plant-based matrices. A dominant inhibitor of zinc absorption is phytate through the formation of poorly soluble complexes [122]. In phytate-rich diets, pH-dependent complexes between zinc and phytate can be formed and contribute to reduced zinc bioavailability. However, this effect is weaker and less consistent than the calcium–phytate–zinc interaction according to literature data [123]. Calcium–zinc interactions appear to be dose, source, and matrix dependent. In a human study, high calcium intake of approximately 1340 mg/day reduced net zinc absorption and zinc balance, and a related single-meal experiment reported an approximately 50% reduction in zinc absorption when calcium was co-administered with the meal [124]. However, other studies have not consistently confirmed this effect, indicating that calcium-mediated inhibition of zinc absorption depends on dietary context, calcium source, phytate level, and zinc adequacy [125].

Phytic acid (PA) acts via a strong mineral chelation mechanism. At the intestinal pH of 6.5 to 7, it forms insoluble, stable complexes with iron and zinc (phytates), preventing their absorption [126]. EFSA summarizes WHO/FAO categories: phytate:zinc ratio <5 = high absorption efficiency; 5–15 = moderate; >15 = low. It also notes that the phytate content of the diet has a major effect on zinc availability [127]. These findings demonstrate a mathematically nonlinear relationship highlighting the stoichiometry of phytic acid–zinc chelation complexes: phytic acid’s six phosphate groups can chelate 2–4 divalent cations (Zn^2+^, Ca^2+^, Mg^2+^) in competitive binding, with relative affinities Zn^2+^ > Ca^2+^ > Mg^2+^ [90,128]. Table S2 (Supplementary Materials) summarizes several studies for on zinc bioavailability by matrix type, phytic acid (PA) content, and PA:Zn molar ratio, while in Table 2 the most recent human trial results on zinc bioavailability are reported.

#### 3.3.3. Calcium

Antagonistic food matrices have a significant influence on calcium bioavailability. Across multiple studies, calcium solubility and absorption are inhibited by PA and oxalic acid (OA) via the formation of insoluble Ca-phytate and Ca-oxalate complexes [108,133]. When these antagonists are present in a food matrix, large reductions in both solubility and cellular uptake occur. OA markedly reduces calcium solubility and permeability by forming insoluble calcium oxalate; standard calcium salts often fall to low permeability levels (<35%) in high-OA conditions [134]. This was verified also by evidence that PA and OA sharply decrease calcium transport in Caco-2 models for both inorganic calcium and peptide–calcium chelates [135]. Both PA and OA significantly reduce calcium absorption rates of CaCl_2_ and peptide-bound calcium; OA has the stronger effect, reducing absorption by 45–57% for CaCl_2_ and 38–45% for peptide complexes [136]. Several studies show that removing, degrading, or counteracting phytic/oxalic acids leads to substantial increases, frequently reaching 50–65%. Fermentation reduces PA/OA, increasing Ca availability by 5–17% and often pushing final availability into the 40–60% range depending on the matrix [137,138]. Casein and peptide-based calcium complexes retain ~50% soluble calcium even in the presence of PA/OA, indicating high antagonist resistance and bioaccessibility potential [139]. In another study, removal of phytic acid in high-protein soymilk increased calcium bioaccessibility by 31% [140]. Recently, it was also shown that isolated hydrolyzed tilapia peptides could enhance calcium transport in Caco-2 cells, reporting increases up to 202 ± 12% compared to control samples [141].

Calcium absorption is also affected by the phosphate and polyphenols content of food. In vitro digestion showed phosphate, palmitic acid, micellar casein, and phytate all reduce calcium solubility in the intestinal phase by forming insoluble complexes [142]. High phosphorus intake and imbalanced calcium:phosphorus ratios (below 0.5–1 or above 1.5–1) can disrupt calcium homeostasis and provoke hormonal responses (PTH, FGF-23), leading to changes in bone structure and function [143]. In leafy vegetables several constituents, including tannins, collectively explained 45% inhibition of Ca bioavailability [144,145]. A review on calcium bioavailability in legumes concluded that polyphenols are one of the main antinutritional factors negatively affecting calcium bioaccessibility in legumes [146].

#### 3.3.4. Plant-Derived Enhancers

Plant-derived enhancers for mineral bioavailability offer promising strategies for functional food development through multiple mechanisms. The presence of polyphenols was found to have a two-way effect on mineral bioavailability. Low polyphenol content can improve the mineral bioavailability by reducing ferric (Fe^3+^) to ferrous (Fe^2+^) iron and forming soluble chelation complexes. At low quercetin:iron ratios (0.5:1), quercetin increased ferritin formation, indicating enhanced iron uptake. This enhancement disappeared or reversed at higher ratios [101]. Another study on the functionality of berries’ anthocyanins, it was shown that they may improve iron absorption in the duodenum or proximal jejunum by converting ferric to ferrous ions [147].

The incorporation of nicotianamine and plant-based compounds into functional foods enhanced iron, zinc and calcium bioavailability (Table 3). The main mechanisms of enhancement are: (1) forming of stable complexes with (Fe^2+^) iron, facilitating iron uptake.; and (2) formation of soluble, high affinity 1:1 nicotianamine–metal chelation complexes [148,149].

Organic acids, such as citric/malic acids, create stable complexes with multiple metals at moderate pH, enhancing their solubilization and potentially improving their bioavailability [150,151,152].

**Table 3:** Enhancement of mineral bioavailability.

Enhancer Type	Mechanism	Minerals Improved	Reference
Nicotianamine (NA)	Chelates Fe^2+^/Zn^2+^, improves transport and solubility	Fe, Zn	[153,154,155,156]
Organic acids (ascorbic, citric, etc.)	Reduce Fe^3+^ → Fe^2+^, form soluble chelates	Fe, Zn	[150,157]
Berry polyphenols	Reduce ferric to ferrous iron	Fe	[147,158]
Plant-derived peptides	Metal binding, protect minerals during digestion	Fe, Zn, Ca	[159,160]
Moringa leaf hydrolysate	Enhances pH-dependent Ca solubility	Ca	

## 4. Gastrointestinal Interactions and Bioaccessibility Modulation

### 4.1. Oral Processing and Gastric Digestion

The first critical stage regulating bioaccessibility and eventually bioavailability is oral processing. Mechanical disruption of plant tissues and reduction in particle size accompanied by increase in surface area are the main phenomena taking place during mastication. These facilitate the release of intracellular compounds such as polyphenols, carotenoids, lipids and minerals [161,162]. This process is further modified by saliva, which hydrates and dilutes the bolus and initiates enzymatic hydrolysis (a-amylase and lingual lipase) [163]. At the same time, saliva can also act as an inhibitor of bioaccessibility. Phenomena such as mucin binding, protein–polyphenol interactions and dilution can exert inhibitory effects on releasing nutrients from the matrix [164]. Overall, oral processing should be considered an integral part of matrix-driven bioaccessibility because it determines the initial physical disintegration and biochemical environment that shape subsequent gastric and intestinal release [165].

Ingested bioactive compounds encounter variable physicochemical conditions during gastrointestinal digestion. Gastric pH is influenced by food composition, while returning to basal level can take up to 4 h. Fasting gastric pH ranges between 1 and 3 [166]. Protein digestion elevates gastric pH depending on protein concentration, with high protein content resulting in longer time to return to basal levels [167]. Higher carbohydrate content in meals requires around 50 min for the pH to return to baseline [168]. High fat meals affect gastric pH more profoundly than low fat, both in value and time to return to basal level [169,170].

Different food matrices can affect the fate of vitamin D_3_ during digestion. Implementation of the INFOGEST protocol for in vitro digestion showed that vitamin D_3_ degradation depends on gastric pH, with pH 1 resulting in 58% degradation, while pH 7 in 44%. Gastric content varied between 39.87 to 47.14 μg/mL, while resulting intestinal bioaccessibility index ranged from 0.54 to 0.74 across gastric pH 1–5 conditions [171]. These observations indicate the significance of taking into account the stability and solubility of target bioactives and how they are affected by pH variation when aiming to design functional foods.

pH effect on bioactives is highly dependent on food matrices and their physicochemical properties. “Matrix effect” is a term used to explain the influence of components and structure of foods on bioactive behavior. The conditions encountered in the GIT during digestion, including enzymatic accessibility and pH variation, are interconnected with the final bioaccessibility and bioavailability of bioactives. The matrix effect can influence the digestion response, and therefore contribute to bioaccessibility variations observed for different food matrices [172,173].

### 4.2. Small Intestinal Digestion and Micelle Formation

Bioactive release and bioaccessibility depend on digestive enzymatic activity. The type of food matrix, structure and interactions between different components, such as adsorption on lipid droplet surfaces, can affect enzymatic activity and lipid digestion with a corresponding effect on bioactive compound bioaccessibility. Lipid hydrolysis is affected by the structure of the matrix, with encapsulated lipid droplets having the lower percentage of released free fatty acids compared to free droplets during intestinal digestion [174].

During lipolysis, triglycerides are hydrolyzed into free fatty acids (FFAs) and monoacylglycerols (MAGs). FFAs have low aqueous solubility and tend to accumulate at the lipid–water interface, where evidence shows that the poorly soluble FFAs can precipitate with minerals [175,176]. Furthermore, mineral precipitation with bile acid–FFA complexes during micellization has been reported in the literature, which contribute to decreased mineral bioavailability [177].

The influence of digestive enzymes on food nutrient hydrolysis is of great interest and has been addressed thoroughly in literature. In cases such as emulsions, hydrolysis may be limited due to stereochemical inhibitions and enzyme displacement. The final bioaccessibility of bioactive compounds, such as phenolics, depends on the ability to be released from the matrix as well as physicochemical interactions during digestion. The polarity and chemical structure of phenolics influence their bioaccessibility. Lipophilic phenolic compounds, such as tocopherols, are incorporated into mixed micelles [178]; hydrophilics, such as anthocyanins, remain in the aqueous phase, while some fractions are bound to other compounds [179]. The final bioaccessible fraction depends on the partitioning of bioactives between these different phases.

Lipophilic bioactive compounds bioavailability is affected by lipid digestion. Encapsulated coenzyme Q10 incorporated in nanoemulsions exhibited increased bioavailability compared to powdered form and medium-chain triglyceride oil [180]. Advanced formulations of coenzyme Q10 have resulted in 1.8-to-2.8-fold increases in bioavailability. Nanoemulsions showed higher bioavailability compared to oil dispersions, which was attributed to the higher lipolysis rate observed for the nanoemulsion due to the larger interfacial area of nanoemulsion droplets [181].

Structural differences such as those found in emulsion-filled hydrogels compared to simple emulsions can affect the bioaccessibility of bioactive compounds. Curcumin bioaccessibility was found to depend on pectin percentage in emulsion-filled hydrogel systems, with increasing percentages showing a reverse effect on bioaccessibility [182]. Increased stability of filled hydrogels can offer greater bioaccessibility for lipophilic compounds such as β-carotene [183].

### 4.3. Colonic Metabolism and Microbiome Transformation

Bioactive metabolism and overall bioavailability are closely linked to gut microbiota. Gut microbiota act upon many bioactives, leading to transformation, resulting in either enhancement or reduction in their biological effectiveness. In the case of polyphenols, though most digestion and absorption of lipids, proteins, starch and carbohydrates take place in the small intestine, colon microbial-mediated metabolism is also of great importance [184,185]. Altering microbiota can affect the bioactive release in the large intestine. When gut microbiota are suppressed, changes in metabolism and pharmacokinetics of flavonoids were observed, indicating the crucial role of microbial metabolism in the bioavailability of bioactives [186].

Gut microbiota and plant-derived bioactives are interconnected. Ingested bioactives can affect the growth and population of each bacteria family present in the large intestine. Polyphenols aid in the growth of Bifidobacteria, while tannins also promote the growth of Lactobacillus [187]. Dietary polyphenols and flavonoids act as substrates for gut microbiota, which then transform them in active metabolites with antioxidant effects [188]. The effect of polyphenols on gut microbiota is dose dependent: when the polyphenol supplemental dose was less than the turning point, there was an increased but limited effect. When the polyphenol supplemental dose was over the turning point dose value, the beneficial responses became inert [189].

Mineral bioavailability is also partly regulated by gut microbiota, which influences their solubility and release from the food matrix. Minerals are unaffected by direct microbial metabolism. Magnesium has been found to increase beneficial gut microbiota populations, such as *Bifidobacterium* related to metabolic homeostasis [190], and *Carnobacterium maltaromaticum* and *Faecalibacterium prausnitzii* associated with vitamin D synthesis to inhibit colorectal cancer [191]. The modulation of gut microbiota by Shouhui Tongbian capsules, a traditional Chinese medicine, resulted in an increase in calcium absorption. This increase was attributed to acidifying the intestinal microenvironment due to the enrichment of bacterial populations from Shouhui Tongbian and intestinal barrier restoration [192].

Formulations that could protect bioactive compounds during gastrointestinal digestion could potentially increase their beneficial effects. Encapsulated resveratrol in casein nanoparticles resulted in approximately 10 times higher bioavailability than resveratrol solution [193], while zein-based nanoparticles increase resveratrol bioavailability by 50% compared to the control [194]. Yogurt fortified with calcium and magnesium encapsulated in brown rice protein increased their bioaccessibility from 53.72 to 57.33% and 10.72 to 15.60%, respectively. Further improvement was observed when brown rice protein formed a conjugate with microcrystalline cellulose by the Maillard reaction, with bioaccessibility reaching 64.40% for calcium and 31.14% for magnesium [195]. These results show that nanosystems can assist in increasing the stability and the bioavailable fraction of bioactives, leading to a shift in the amount reaching colonic microbiota [196].

### 4.4. Host-Related Factors and Individual Variability

Variability in the observed bioaccessibility and bioavailability of nutrients is also a result of host-related factors, including age, gender, gut microbiota, health status and genetics. These factors could affect bioactive bioaccessibility and bioavailability not only due to individual differences, but also due to interactions with external factors, such as structure of bioactive, food composition and processing. Iron deficiency has been linked to reduced levels of hydrochloric acid in gastric secretions [197]. It has also been associated to *Helicobacter pylori* infection, which influences the gastric environment [198]. Gut microbiota individual variability is a contributing factor to bioavailability of nutrients. Phytases are enzymes produced by intestinal bacteria that act on phytates from plant-derived ingredients, which chelate micronutrients and therefore reduce their bioavailability. Phytase expression and activity depends on the population combination of intestinal bacteria [199]. These differences indicate the substantial effect of individual variability in gut microbiota and pH level on the absorption of nutrients.

Health and nutritional status are important aspects of nutrient absorption. Geriatric hospitalized patients showed a decrease in iron absorption depending on the C-reactive protein concentration [200]. Micronutrient absorption is impaired in patients that have undergone bariatric surgery, even 2 years after surgery [201]. Crohn’s disease could also result in malabsorption of nutrients with the effect depending on disease status [202]. The need for effective carriers and functional foods is therefore highly important to address reduced nutrient absorption depending on medical conditions.

Age should be considered when nutrient absorption is discussed. Gastrointestinal tract physiological functions and microbiota differ between infants, adults and elderly [203]. Nutrient absorption, such as for vitamin D, vitamin B12 and calcium, reduces with age, which is attributed to reduced enzyme activity and acid secretions [204].

Differences in bioavailability of nutrients have been observed depending on gender. Carotenoid concentration in women was higher compared to men, even if the dietary intake was lower [205]. Polyphenol bioavailability is also influenced by gender, with naringenin having greater bioavailability in women, while eriodictyol and homoerodyctiol are greater in men. Differences in polyphenol concentration could indicate differences in regulation of metabolic pathways for these nutrients that are dependent on gender [206]. Furthermore, microbiota composition dependent on gender has been linked to differences in polyphenol absorption [207]. Health status and gender are also interdependent, as lower prevalence of chronic kidney disease in women has been associated with higher carotenoid intake, though the same correlation was not found in men [208].

Genetic variability is also a factor which could influence bioavailability of nutrients. Two nonsynonymous genetic variants that are associated with β-carotene metabolism were shown to affect vitamin A status [209]. Single nucleotide polymorphism was associated with differences in circulating concentrations of zeaxanthin, lycopene and β-carotene [210]. The differences observed in absorption of bioactives depending on interindividual variability should be taken into account when functional food formulations are developed.

## 5. Functional Food Development Strategies and Matrix-Based Approaches

### 5.1. Rational Food Matrix Design and Optimization

The development of functional foods demands rational optimization of food matrix composition to achieve maximum bioaccessibility of the bioactive substances. Matrix optimization appears to be challenging, as the food matrices can be viewed as parts of the microstructures of the food and can affect post-ingestion nutrient bioavailability and the overall sensory profile of the food. This presents a valuable opportunity to engineer food matrices that enhance both the bioavailability and organoleptic qualities of food, resulting in the formulation of products that have optimal balance between consumer appeal and demonstrable health impacts [211,212].

Targeted matrix structures manipulation can be achieved through specific ingredient selection and processing, which can substantially improve the overall potency of functional foods. This approach relies on the principle that molecular design and processing conditions, i.e., composition, pH, temperature, can regulate the biophysical behavior of lipid assemblies to generate colloidal structures (e.g., micelles, vesicles, nanodiscs, and liquid crystalline phases) that enhance stability, solubility, and ultimately absorption [213,214,215]. For instance, choosing the type of protein and the level of structural adjustment controlled by various methods such as physical, chemical, thermal or enzymatic, can achieve enhanced protein digestibility. This shows that multiple matrix components can be improved at the same time.

### 5.2. Nanoencapsulation and Advanced Delivery Systems

Employing nanoencapsulation and nanoemulsion systems can provide a highly effective way to improve the absorption of bioactive substances in functional foods. Major key factors that affect those transport mechanisms of bioactive compounds include simulated gastrointestinal digestion models, nanoemulsions composition and food matrix properties [216,217]. Components such as minerals, polysaccharides, salts, proteins and surface-active agents adhere to the nanoparticles and cause physical transformations. These interactions induce structural shifts and sequentially alter their bioaccessibility.

To engineer efficient nanoencapsulation systems, it is critical to evaluate the complex interactions among gastrointestinal conditions, nanocarrier materials and components of the food matrix. It is important to consider the advantages and limitations of lipid, surfactant and polymeric-based nanocarriers, since bioaccessibility of encapsulated bioactives can affect oral bioavailability both in a negative or positive way. Before absorption, bioactives must be released from their matrix nanocarriers, a process dependent on texture, processing techniques, matrix aggregation state, and digestive fluid properties including pH, viscosity, ionic strength and enzymatic activity.

### 5.3. Strategic Fortification and Bioavailability Enhancement

**Figure 1:**
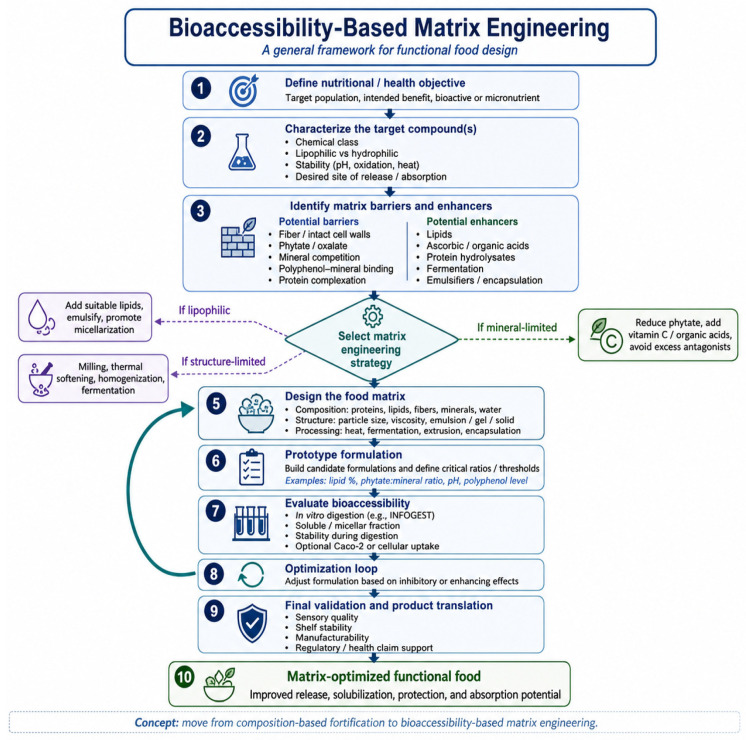
Flowchart for bioaccessibility-based matrix engineering.

Functional food enhancement must consider the complexity of interaction between fortifying agents and natural components of food matrices. The effectiveness of functional foods can be significantly increased through strategic selection of fortifying agents that stimulates the release of target bioactive substances. Optimizing the micronutrient bioaccessibility, for example, requires thorough understanding of which matrix components act as barriers and which as promoters of metal absorption, as described previously. Studies on commercial products have shown that well-engineered fortification matrices boost mineral bioaccessibility in comparison with simple approaches (Table 4). Thus, investing in advanced research on matrix design is a cost-effective strategy for product optimization. A proposed step-by-step flowchart for engineering enhanced matrices for improved bioaccessibility is shown in Figure 1.

### 5.4. Example of Practical Application

A *hypothetical* practical application of matrix-based functional food design is the development of a fermented oat–carrot–citrus emulsion beverage fortified with iron, zinc, β-carotene, and curcumin. The product is not designed by asking, “How much iron, zinc, carotenoid, and curcumin can be added?”; rather, it is designed by asking, “Which matrix architecture allows these compounds to be released, solubilized, protected, and absorbed?”. That distinction fits the central argument *that functional food development should move from composition-based fortification to bioaccessibility-based matrix engineering*.

In this model system, the oat matrix provides consumer familiarity and plant-based positioning but also introduces phytate as a major mineral antagonist. Therefore, fermentation with lactic acid bacteria and/or phytase treatment is applied before mineral fortification to reduce phytate and improve iron and zinc solubility. The carotenoid-rich carrot fraction is thermally softened and homogenized to disrupt plant cell walls, while β-carotene and curcumin are incorporated into a structured oil-in-water emulsion containing approximately 6–8% lipid to promote mixed micelle formation during intestinal digestion. Vitamin C from citrus or acerola is included to enhance non-heme iron bioaccessibility, while high-tannin ingredients, excessive calcium fortification, and high-viscosity fiber enrichment are avoided because they may antagonize mineral uptake. This example illustrates that functional efficacy depends not only on the amount of bioactive compounds added but also on the rational control of matrix structure, lipid phase behavior, phytate:mineral ratios, polyphenol concentration, and gastrointestinal release mechanisms. The final formulation should therefore be validated using standardized in vitro digestion, such as the INFOGEST protocol, followed by quantification of soluble or micellarized fractions of iron, zinc, β-carotene, and curcumin.

## 6. Conclusions and Future Perspectives

Bioaccessibility and bioavailability of bioactive compounds are strongly determined by the food matrix, which in recent years has emerged as a critical factor in determining the nutritional quality and health beneficial effects of foods and functional foods.

Future functional food development must embrace matrix-informed approaches that optimize food structure and composition to enhance bioactive bioaccessibility while maintaining sensory appeal and shelf stability. The emerging non-thermal processing technologies, nanoencapsulation strategies, and rational matrix design approaches offer exciting opportunities to develop functional foods with substantially improved efficacy compared to current products [46]. As the scientific understanding of matrix–bioactive interactions continues to deepen, the food industry will increasingly employ these insights to create products that deliver meaningful nutritional and health benefits to consumers.

The integration of in vitro bioaccessibility assessment, computational modeling, and personalized nutrition approaches represents a promising frontier for functional food development. Understanding the probabilistic nature of matrix effects and their complex interactions across populations suggests that future functional foods may be designed for specific demographic groups or tailored to individual genetic and microbiota profiles. Such approaches would substantially increase the likelihood that functional foods deliver their intended health benefits, transforming them from aspirational products to scientifically validated therapeutic foods [224,225,226].

Specific research goals should include the following:

Shift from descriptive to predictive bioavailability/bioaccessibility approaches. Current knowledge and tools of artificial intelligence and mechanistic modeling allow the development of predictive models that will be able to estimate bioavailability from matrix composition, structural properties and processing conditions.

To achieve the above-mentioned goal, it is necessary to understand complex, non-linear and multifactor interactions, not just a single factor effect. Most studies focus on simple interactions (e.g., phytic acid–iron, etc.), while the real food matrix is more complex, involving lipid–protein–fiber–bioactive–mineral interactions. This approach will allow researchers and practitioners to establish quantitative design rules for functional foods by identifying critical threshold values, such as phytic acid to zinc ratio, etc.

Furthermore, future research should ensure translational relevance to real diets. Many innovations (e.g., encapsulation, emulsions, etc.) are validated in lab-scale experiments. The next step should examine how these systems behave in whole foods, mixed meals and real dietary patterns, either in animal models or human trials. In addition, research should examine not just the composition of the matrix but also the structure. There is insufficient focus on food structure, i.e., cell wall structure, mechanical properties, particle size, and how these affect the bioavailability of nutrients.

## Figures and Tables

**Table 1:** Food matrix effects on iron absorption—human studies (2015–2025).

Matrix Factor	Food Matrix/Test Condition	Measured Effect on Iron Absorption or Solubility	Interpretation	Citation
Phytate	Finger millet meals differing in phytic acid	Low-phytate variety increased iron absorption from 1.3% to 3.7%	Approximately 3-fold increase after lowering phytate	[116]
Phytate in habitual diets	Kuwaiti mixed dishes evaluated for phytate:mineral ratios	Only 13.2% of dishes provided adequate predicted iron bioavailability	Phytate:mineral ratio was the main limiting factor	[117]
Polyphenols/tannins	Blueberries consumed with 5 mg Fe	Iron absorption decreased from 30.2% to 6.8–7.5%	Approximately 75% reduction	[118]
Tea tannins	Tea consumed with Fe-rich porridge vs. 1 h separation	Iron absorption decreased from 5.7% to 3.6% when tea was consumed with the meal; 1 h separation restored absorption to 5.7%	Concurrent tea intake inhibited iron absorption; separation reduced inhibition	[119]
Tannic acid	5 mg Fe + 100 mg tannic acid, fasting state	Iron bioavailability decreased from 25.0% to 16.8%	Approximately 33% reduction	[120]
Pulse polyphenols/tannins	Chickpea-containing human test meals with added tannins	Soluble iron decreased by approximately 28% relative to non-pulse control meals	Tannins reduced soluble iron during digestion	[121]
Calcium	5 mg Fe + 800 mg CaCl_2_, fasting state	Iron absorption was not significantly different from placebo	Calcium effect was minimal under this test condition	[120]

**Table 2:** Recent human trial studies (2020–) on zinc bioavailability by matrix type, phytic acid (PA) content, and PA:Zn molar ratio.

Study (Year)	Matrix Type	PA Content	PA:Zn Ratio	Bioavailability Outcome	Reference
Glycoprotein Zinc randomized crossover trial (RCT) (2024)	Supplement (GPM vs. ZnO)	NA *	NA *	GPM ↑ absorption by 40%	[129]
Biofortified Potatoes (2023)	Whole potatoes	NR **	NR **	Higher total absorbed Zn	[130]
Biofortified Rice RCT (2024)	Mixed meals	Indirect	Determines FAZ (23–31%)	High PA:Zn → low absorption	[131]
Kuwait Dietary Dishes (2023)	Mixed foods	Measured	<5, 5–15, >15	>15 = low absorption	[117]
Irish Women Diet Study (2023)	Habitual diets	NR	Inferred high	High-phytate diets → low Zn status	[132]

*, ** NA: not applicable; NR: not reported. ↑: increase; →: leads to.

**Table 4:** How engineered matrices outperform simple mineral additions.

Engineered Matrix Type	Product Type	Improvement in Bioaccessibility	Citation
Double emulsions	Infant and adult systems	Iron bioaccessibility 39–50%	[218]
Microparticles (spray drying + chilling)	Plant-based yogurt	Iron bioaccessibility 59.5%	[219]
Granulated salts	Leafy vegetables	Fe and Zn bioaccessibility several-fold higher	[220]
Bioprocessed dual-fortified grains	Red rice	Zn bioaccessibility ↑3–7×; Fe ↑2–2.5×	[221]
Nanoparticle minerals	Yogurt	Higher solubility and bioaccessibility vs. micro forms	[222]
Low-phytate product matrix	Plant-based burgers	Fe and Zn bioaccessibility ~100%	[223]
Precision-engineered food matrices	Multiple systems	Improved release and bioavailability	[46]

↑: increase.

## Data Availability

No new data were created or analyzed in this study. Data sharing is not applicable to this article.

## Supplementary Materials

The following supporting information can be downloaded at https://www.mdpi.com/article/10.3390/ijms27125503/s1. References [227,228,229,230,231,232,233,234,235,236,237,238,239,240,241,242,243,244,245,246,247,248] are cited in the supplementary materials.
