# Supplementary material for: Food Matrix Effects on Plant-Derived Bioactive Compounds and Micronutrients: Implications for Functional Food Development
Source: Int J Mol Sci. 2026 Jun 18;27(12):5503. doi: 10.3390/ijms27125503 (PMC13299729; doi:10.3390/ijms27125503)
Supplement: Supplementary file 1 [file ijms-27-05503-s001.zip › ijms-4350844-supplementary.pdf]

Table S1. Effects of food matrix components on the release and bioaccessibility of plant-derived bioactives and micronutrients.

Matrix component / factor	Target compounds	Main mechanism	Effect on bioaccessibility / bioavailability	Quantitative or representative evidence	References*
Phytate-rich matrix	Zinc	Mineral chelation and formation of poorly soluble complexes	Inhibition	Increased phytic acid decreases zinc bioavailability; effect depends on zinc source and phytic acid:zinc molar ratio, with significant impact reported at ratios higher than 1:100	[24–26]
Proteins, general	Polyphenols, carotenoids, minerals	Covalent and non-covalent binding, complex formation, altered solubility and digestibility	Enhancement or inhibition depending on protein and bioactive type	Protein–bioactive complexes may alter solubility, digestibility, and bioavailability; effects are compound-specific	[31,32]
β-lactoglobulin	Carotenoids	Hydrophobic binding and possible transport toward enterocyte brush border	Enhancement	β-lactoglobulin increased carotenoid bioaccessibility after thermal processing by 26–354%, depending on temperature	[33,34]

Matrix component / factor	Target compounds	Main mechanism	Effect on bioaccessibility / bioavailability	Quantitative or representative evidence	References*
β-casein	Green tea catechins	Protein–polyphenol binding and altered release	Mixed; inhibition for EGCG and EGC, enhancement for EC	β-casein reduced EGCG and EGC bioaccessibility, while increasing EC bioaccessibility	[35]
Sodium caseinate	EGCG	Protein-stabilized emulsion and intermolecular interactions improving intestinal permeability	Enhancement	Sodium caseinate-stabilized emulsions increased EGCG bioaccessibility by 64% compared with free EGCG after simulated gastrointestinal digestion	[36,37]
Soy protein	Zinc and iron	Mineral binding, protein–mineral interactions, and possible interference from phytate/protein moieties	Mixed	Soy protein increased zinc bioaccessibility from sorghum and rice by 50% and 90%, respectively, but decreased iron bioaccessibility in both grains	[38,39]
Digested whey proteins / protein hydrolysates	Minerals	Chelation, protection, and carrier-mediated transport	Enhancement	Digested whey proteins improved mineral bioavailability and cellular	[40–42]

Matrix component / factor	Target compounds	Main mechanism	Effect on bioaccessibility / bioavailability	Quantitative or representative evidence	References*
				uptake in Caco-2 models compared with free minerals; protein hydrolysates may increase mineral solubility	
Dietary fiber, general	Polyphenols and other bioactives	Binding to fiber structures and delayed release during digestion	Mainly inhibition, but matrix-dependent	Fiber-bound polyphenols require additional digestive release steps, reducing bioaccessibility compared with unbound forms	[43,44]
Insoluble dietary fiber	Tannins, lignins, polymerized phenolics	Physical entrapment and limited release from the insoluble fraction	Inhibition	Highly polymerized phenolic species associated with insoluble fiber show low bioaccessibility due to limited release from the matrix	[45,46]
Hemicellulose and viscous/protein-rich meals	Polyphenols	Non-covalent sequestration and increased viscosity	Inhibition	Hemicellulose generally reduces polyphenol bioaccessibility; viscous	[47,48]

Matrix component / factor	Target compounds	Main mechanism	Effect on bioaccessibility / bioavailability	Quantitative or representative evidence	References*
				and protein-rich meals may further reduce release	
Soluble fibers, β-glucans, pectins	Polyphenols and carotenoids	Modification of pH, viscosity, microbiota, and micellarization behavior	Enhancement or inhibition depending on fiber type and bioactive	Soluble fibers can enhance bioavailability by modifying intestinal pH and microbiota, while insoluble fibers mainly reduce release through sequestration	[47,48]
Apple fiber and plant cell-wall polysaccharides	Quercetin	Hydrogen bonding and hydrophobic interactions with pectin regions and neutral sugar side chains	Inhibition / altered release	Quercetin shows higher affinity for apple fiber than citrus fiber; FTIR-ATR indicates structural changes after binding	[49,50]
Arabinoxylans / hemicelluloses in grains	Ferulic acid	Strong binding of phenolic acids to grain cell-wall components	Strong inhibition	Ferulic acid bioaccessibility was <1% in intact grain products, whereas free ferulic acid added to flour reached 60% bioaccessibility	[52]

Matrix component / factor	Target compounds	Main mechanism	Effect on bioaccessibility / bioavailability	Quantitative or representative evidence	References*
Lipid content	Curcumin, carotenoids, other lipophilic bioactives	Formation of mixed micelles after lipid digestion	Enhancement when optimized; insufficient or excessive lipid may reduce efficiency	Lipophilic bioactives require adequate lipid for micellar solubilization; carotenoid absorption may require ~3–5 g fat per meal	[58,59]
Higher lipid levels	Curcumin	Lipase-mediated hydrolysis of triglycerides into monoacylglycerols and free fatty acids, followed by mixed micelle formation	Enhancement	Curcumin absorption increased nearly 2.5-fold when consumed with 20% coconut oil	[60,61]
Corn oil addition	β-carotene	Lipid-assisted micellarization	Strong enhancement	β-carotene bioaccessibility reached 93.2% with 10% corn oil	[62]
Lipid type: MCTs vs LCTs	Curcumin, vitamin D, lipophilic bioactives	Fatty acid chain length affects micelle stability, lipolysis, and solubilization capacity	LCTs generally more favorable for large lipophilic molecules	MCTs form less stable mixed micelles than LCTs; longer chains improved curcumin bioaccessibility	[63–66]

Matrix component / factor	Target compounds	Main mechanism	Effect on bioaccessibility / bioavailability	Quantitative or representative evidence	References*
Lipid unsaturation and oil source	Curcumin	Degree of unsaturation affects lipolysis and tissue distribution	Matrix-dependent enhancement	Curcumin in linseed oil produced higher curcumin levels in serum, liver, heart, and brain than coconut or sunflower oil nanoemulsions	[64–67]
Dietary fat level and lipid type	Vitamin D3	Fat-assisted solubilization and micellar incorporation	Enhancement	Vitamin D3 bioavailability increased by 32% with a 30% fat meal compared with minimal lipid; LCTs were more effective than MCTs for intestinal absorption	[68,69]
Lipid-based delivery systems	CoQ10	Nanoemulsion or beverage lipid systems improve dispersion and absorption	Enhancement	CoQ10 bioavailability increased 1.8- to 2.8-fold in lipid-based formulations compared with CoQ10 dissolved in oil alone	[70]

References* numbering refers to main text references

Table S2. Studies on Zinc Bioavailability by Matrix Type, Phytic Acid (PA) Content, and PA:Zn Molar Ratio

Matrix	PA Level	PA:Zn Molar Ratio	Effect on Zinc Bioavailability	Reference
Rice cultivars	Variable PA (0.82–2.62 g/100 g)	Higher PA correlated with higher ratios	Low PA rice (e.g., Bindli) showed higher Zn bioavailability; high PA (PB267) showed lower bioavailability	[1]
Iranian breads (Lavash, Mashini, Sangak, etc.)	Moderate–high	Flour: ~19; Breads: ~9	Baggett & Roghani breads showed good bioavailability (50–55%); others moderate (30–35%); flour low (10–15%)	[2]
Yogurt + cereals	Low–moderate	0.83–3.89	PA:Zn <5 suggests >50% bioavailability	[3]
Malaysian foods (rice, wheat, grains)	Many high-PA foods	Some >15	High phytate content likely impairs Zn bioavailability	[4]
Wheat (biofortified vs. high-PA)	Varies; low-PA mutants	PA:Zn <15 in best cases	Strong inverse relationship: PA:Zn negatively correlates with Zn uptake (r = –0.76 to –0.94)	[5]
Wheat flour	272mg/100gr	PA:Zn>15	Low Zn bioavailability (10-15%)	[6]

Matrix	PA Level	PA:Zn Molar Ratio	Effect on Zinc Bioavailability	Reference
Rice (processing effects)	Moderate PA	Not always correlated	Milling reduces Zn; PA reduction improves Fe more than Zn	[7]
Vegetarian meals (mixed matrices)	High	Variable	Bioavailable Zn predicted mainly by IP3, IP5 breakdown products and Zn content	[8]
Brown rice (soaking, germination, fermentation)	Initial high PA; reduced by processing	Reduced after fermentation	Large PA reductions did not always increase in vitro Zn solubility	[9]
Infant cereals	High PA and Ca	PA:Zn = 22–75	High PA+Ca substantially reduce Zn bioavailability; Ca amplifies PA inhibition	[10]
Low-phytate cereals (maize, barley, rice)	Low PA	Much lower ratios	Zn absorption increases dramatically in low-PA variants (maize: from ~21–33% to 47–52%)	[11]
Beans (various cooking methods)	Variable	Not given	Up to 20% Zn bioavailable; PA correlations weak	[12]
Fermented cassava (plant-based diet)	PA reduced 90%	Lower PA:Zn	Fermentation sharply improved Zn absorption	[13]

Matrix	PA Level	PA:Zn Molar Ratio	Effect on Zinc Bioavailability	Reference
Chinese diet in women	Moderate	PA:Zn ≈ 3	Zn absorption ~36–38%—PA and Ca not major inhibitors in this diet	[14]
Millet porridge (children)	High	PA:Zn = 7.7	Phytase addition increased FAZ from 9.5% to 16%	[15]
Sourdough breads	PA reduced by fermentation	Lower ratios	Improved Zn bioavailability index	[16]
Fortified rice	Moderate	PA:Zn = 12	Biofortified and fortified rice had similar Zn absorption	[17]
High-PA tortillas (maize)	High	23–26 in WT	Low-PA hybrids: much higher FAZ (0.28–0.38 vs 0.13–0.15)	[18]
Thai rice (foliar Zn)	Reduced PA:Zn with Zn foliar application	Lower ratios	Higher grain Zn and lower PA:Zn improve bioavailability	[19]
China: 60 common foods	Many high-PA foods	31 foods >15 ratio	High PA:Zn >15 predicts poor bioavailability	[20]
Plantain flour fortified with okra seed	Moderate	PA:Zn below critical values	Fortification increased Zn and lowered PA:Zn → better bioavailability	[21]
Breakfast wheat flakes	High	PA:Zn=35	48% reduction in phytate content and a 1.1-fold increase in fractional Zn absorption	[22]

(1) Kumar, A.; Lal, M. K.; Kar, S. S.; Nayak, L.; Ngangkham, U.; Samantaray, S.; Sharma, S. G. Bioavailability of Iron and Zinc as Affected by Phytic Acid Content in Rice Grain. *J. Food Biochem.* **2017**, *41* (6), e12413. https://doi.org/10.1111/JFBC.12413;ISSUE:ISSUE:DOI.

(2) Gargari, B. P.; Mahboob, S.; Razavieh, S. V. Content of Phytic Acid and Its Mole Ratio to Zinc in Flour and Breads Consumed in Tabriz, Iran. *Food Chem.* **2007**, *100* (3), 1115–1119. https://doi.org/10.1016/J.FOODCHEM.2005.11.018.

(3) del Carmen Borelli, M. F.; Ramón, A. N.; de la Vega, S. M. Calcium-Phytate-Zinc Interaction in Yogurt with Cereals[Interacción Calcio-Fitato-Cinc En Yogures Con Cereales]. *Revista Espanola de Nutricion Comunitaria* **2007**, *13* (1), 26–29.

(4) Norhaizan, M. E.; Nor Faizadatul Ain, A. W. Determination of Phytate, Iron, Zinc, Calcium Contents and Their Molar Ratios in Commonly Consumed Raw and Prepared Food in Malaysia. *Malays. J. Nutr.* **2009**, *15* (2), 213–222.

(5) Salunke, R.; Rawat, N.; Tiwari, V. K.; Neelam, K.; Randhawa, G. S.; Dhaliwal, H. S.; Roy, P. Determination of Bioavailable-Zinc from Biofortified Wheat Using a Coupled in Vitro Digestion/Caco-2 Reporter-Gene Based Assay. *Journal of Food Composition and Analysis* **2012**, *25* (2), 149–159. https://doi.org/10.1016/J.JFCA.2011.09.006.

(6) Wang, M.; Kong, F.; Liu, R.; Fan, Q.; Zhang, X. Zinc in Wheat Grain, Processing, and Food. *Front. Nutr.* **2020**, *7*, 546141. https://doi.org/10.3389/FNUT.2020.00124/TEXT.

(7) Kumar, A.; Lal, M. K.; Sahoo, S. K.; Dash, G. K.; Sahoo, U.; Behera, B.; Nayak, L.; Bagchi, T. B. The Diversity of Phytic Acid Content and Grain Processing Play Decisive Role on Minerals Bioavailability in Rice. *Journal of Food Composition and Analysis* **2023**, *115*, 105032. https://doi.org/10.1016/J.JFCA.2022.105032.

(8) Chiplonkar, S. A.; Agte, V. V. Predicting Bioavailable Zinc from Lower Phytate Forms, Folic Acid and Their Interactions with Zinc in Vegetarian Meals. *J. Am. Coll. Nutr.* **2006**, *25* (1), 26–33. https://doi.org/10.1080/07315724.2006.10719511.

(9) Liang, J.; Han, B. Z.; Nout, M. J. R.; Hamer, R. J. Effects of Soaking, Germination and Fermentation on Phytic Acid, Total and in Vitro Soluble Zinc in Brown Rice. *Food Chem.* **2008**, *110* (4), 821–828. https://doi.org/10.1016/J.FOODCHEM.2008.02.064.

(10) Bertinato, J.; Griffin, P.; Huliganga, E.; Matias, F. M. G.; Dam, D.; Brooks, S. P. J. Calcium Exacerbates the Inhibitory Effects of Phytic Acid on Zinc Bioavailability in Rats. *Journal of Trace Elements in Medicine and Biology* **2020**, *62*, 126643. https://doi.org/10.1016/J.JTEMB.2020.126643.

(11) Lönnerdal, B.; Mendoza, C.; Brown, K. H.; Rutger, J. N.; Raboy, V. Zinc Absorption from Low Phytic Acid Genotypes of Maize (Zea Mays L.), Barley (Hordeum Vulgare L.), and Rice (Oryza Sativa L.) Assessed in a Suckling Rat Pup Model. *J. Agric. Food Chem.* **2011**, *59* (9), 4755–4762. https://doi.org/10.1021/JF1043663.

(12) Huertas, R.; William Allwood, J.; Hancock, R. D.; Stewart, D. Iron and Zinc Bioavailability in Common Bean (Phaseolus Vulgaris) Is Dependent on Chemical Composition and Cooking Method. *Food Chem.* **2022**, *387*, 132900. https://doi.org/10.1016/J.FOODCHEM.2022.132900.

(13) Lazarte, C. E.; Vargas, M.; Granfeldt, Y. Zinc Bioavailability in Rats Fed a Plant-Based Diet: A Study of Fermentation and Zinc Supplementation. *Food Nutr. Res.* **2015**, *59*. https://doi.org/10.3402/FNR.V59.27796.

(14) Yang, L.; Yang, X.; Piao, J.; Tian, Y.; Li, P.; Wang, Y.; Wang, J. Studies on Zinc Bioavailability from a Representative Diet in Chinese Urban Women of Childbearing Age Using a Double Label Stable Isotope Technique. *Journal of Trace Elements in Medicine and Biology* **2005**, *19* (2–3), 159–164. https://doi.org/10.1016/J.JTEMB.2005.09.001.

(15) Brnić, M.; Hurrell, R. F.; Songré-Ouattara, L. T.; Diawara, B.; Kalmogho-Zan, A.; Tapsoba, C.; Zeder, C.; Wegmüller, R. Effect of Phytase on Zinc Absorption from a Millet-Based Porridge Fed to Young Burkinabe Children. *European Journal of Clinical Nutrition 2017 71:1* **2016**, *71* (1), 137–141. https://doi.org/10.1038/ejcn.2016.199.

(16) Najafi, M. A.; Rezaei, K.; Safari, M.; Razavi, S. H. Use of Sourdough to Reduce Phytic Acid and Improve Zinc Bioavailability of a Traditional Flat Bread (Sangak) from Iran. *Food Science and Biotechnology 2012 21:1* **2012**, *21* (1), 51–57. https://doi.org/10.1007/S10068-012-0007-3.

(17) Brnić, M.; Wegmüller, R.; Melse-Boonstra, A.; Stomph, T. J.; Zeder, C.; Tay, F. M.; Hurrell, R. F. Zinc Absorption by Adults Is Similar from Intrinsically Labeled Zinc-Biofortified Rice and from Rice Fortified with Labeled Zinc Sulfate. *J. Nutr.* **2016**, *146* (1), 76–80. https://doi.org/10.3945/JN.115.213421.

(18) Hambidge, K. M.; Huffer, J. W.; Raboy, V.; Grunwald, G. K.; Westcott, J. L.; Sian, L.; Miller, L. V.; Dorsch, J. A.; Krebs, N. F. Zinc Absorption from Low-Phytate Hybrids of Maize and Their Wild-Type Isohybrids. *Am. J. Clin. Nutr.* **2004**, *79* (6), 1053–1059. https://doi.org/10.1093/AJCN/79.6.1053.

(19) Bodeerath, S.; Jumrus, S.; Veeradittakit, J.; Utasee, S.; Jamjod, S.; Prom-u-Thai, C. Variation of Grain Zinc, Phytate Concentration and Phytate : Zn Molar Ratio in Unpolished and Polished Rice Affected by Foliar Zinc Application among Thai Rice Varieties. *Plant Prod. Sci.* **2024**, *27* (4), 304–319. https://doi.org/10.1080/1343943X.2024.2412867.

(20) Ma, G.; Jin, Y.; Piao, J.; Kok, F.; Guusje, B.; Jacobsen, E. Phytate, Calcium, Iron, and Zinc Contents and Their Molar Ratios in Foods Commonly Consumed in China. *J. Agric. Food Chem.* **2005**, *53* (26), 10285–10290. https://doi.org/10.1021/JF052051R.

(21) Adetuyi, F. O.; Adelabu, H. A. Impact of Okra (Abelmoschus Esculentus) Seed Flour on Nutrients, Functional Properties and Zinc Bioavailability of Plantain Flour. *Malays. J. Nutr.* **2011**, *17* (3), 359–366.

(22) Huyskens, M.; Lemmens, E.; Abou-Zeid, L.; Hobin, K.; Balsiger, L. M.; Vanhaecke, F.; Verbeke, K.; Smolders, E.; Delcour, J. A. Zinc Absorption from Breakfast Flakes Produced from Sprouted or Hydrothermally Processed Wheat: A Randomized Cross-over Human Intervention Study. *Food Funct.* **2025**, *16* (24), 9377–9389. https://doi.org/10.1039/D5FO03650J.
